# EYE TRACKING TO SUPPORT ASSESSMENT OF PATIENTS WITH PROLONGED DISORDER OF CONSCIOUSNESS: A CASE SERIES

**DOI:** 10.2340/jrm.v57.41324

**Published:** 2025-01-03

**Authors:** Jan JOHANSSON, Marika MÖLLER, Kristina FRANZON, Jonas STENBERG, Alison K. GODBOLT

**Affiliations:** 1 Department of Clinical Neuroscience, Karolinska Institutet, Stockholm, Sweden; 2 Department of Clinical Sciences, Division of Rehabilitation Medicine, Karolinska Institutet, Danderyd Hospital, Stockholm, Sweden; 3 Department of Rehabilitation Medicine, Danderyd Hospital, Stockholm, Sweden; 4 Norwegian University of Science and Technology, Department of Neuromedicine and Movement Science, Trondheim, Norway

**Keywords:** severe brain injury, prolonged disorders of consciousness, Coma Recovery Scale Revised, eye tracking

## Abstract

**Objective:**

To investigate if eye tracking can support detection of covert voluntary eye movements and to compare these findings with a simultaneously performed clinical assessment according to the Coma Recovery Scale manual regarding visual stimuli.

**Design:**

Observational case series.

**Subjects:**

Twelve outpatients with prolonged disorders of consciousness recruited from the rehabilitation clinic of a regional rehabilitation unit.

**Method:**

Eye movements were recorded with a wearable eye tracker while performing 4 test items from the Coma Recovery Scale Revised. The clinical assessment and recorded eye movement responses were analysed for agreement.

**Results:**

Response data was obtained from 238 out of 288 trials. Eye-tracking data were obtained in median 89.6% of the trials (37.5–100%). The eye tracking assessment judged a significantly higher percentage of trials as a response (46.2%) compared with the clinical assessment (18.1%), mainly in test items “visual pursuit” and “visual fixation”.

**Conclusion:**

Eye tracking showed potential to be more effective in the detection of putative voluntary eye movements compared with conventional examination. Based on the findings in this and previous studies, eye tracking may serve as a useful complementary tool when examining patients with prolonged disorders of consciousness.

## LAY ABSTRACT

After a severe brain injury, most patients gradually regain consciousness over days or weeks. However, a minority of patients remain as what appears to be patients with disordered consciousness for more than 1 month. For prognosis and clinical decisions, it is relevant to assess whether there are behavioural signs of consciousness. The assessment is done with the support of behavioural assessment scales such as the Coma Recovery Scale Revised but it is challenging and they have high rates of misdiagnosis. The purpose of this study was to investigate if eye tracking technology contributes to the detection of voluntary eye movements. The findings from visually based Coma Recovery Scale Revised items performed according to the Coma Recovery Scale manual were compared with assessment based on recorded eye movement data. Eye tracking showed potential in detection of eye movements. Based on the findings in this and previous studies, eye tracking may serve as a useful tool that may complement a clinical observation.

After a severe brain injury, most patients gradually regain consciousness over days or weeks. However, a minority of patients remain in what appears to be an unresponsive state for more than 1 month, a condition usually referred to as a prolonged disorders of consciousness (PDOC) (1). PDOC encompasses unresponsive wakefulness syndrome (UWS)/vegetative state, in which patients show no behavioural signs of consciousness, and minimally conscious state (MCS), in which patients show inconsistent signs of consciousness (2). As prognosis is better for patients in MCS compared with UWS (3), differentiation of these categories is relevant for clinical decisions, rehabilitation planning, and information-giving to family and caregivers (4).

It has been established that differentiation between UWS and MCS is challenging, with high rates of misdiagnosis (5, 6). Behavioural assessment scales are important tools in the differential diagnosis of PDOC (5). The Coma Recovery Scale Revised (CRS-R) is the most established standardized scale (7), and its use is recommended in international guidelines (1) as it allows detection of signs of consciousness for patients in whom these may be missed with standard neurological examination (5, 6). The CRS-R is designed to detect conscious responses, whilst at the same time ensuring that random, non-stimuli-related responses are not misinterpreted as being conscious. As such, criteria are of necessity of an “all or none” quality, with clear definitions of clinically certain responses, for example visual pursuit movements defined in terms of degrees of movement. The clinical reality is that there are a range of responses that do not meet CRS-R criteria, from clearly absent responses to responses not fully meeting CRS-R criteria. Evaluation of these “nearly” responses could be improved with increased objectivity and data from biometric devices.

The behavioural responses in the CRS-R include observation of eye movements in response to an instruction or a presented stimulus. Eye movement control involves widespread brain networks (8, 9). Damage to these networks is common after severe brain injury and may significantly affect eye movements, which may be subtle, delayed, and imprecise and therefore challenging to assess. Eye-tracking technology could potentially contribute to the detection of conscious responses, which may be difficult to detect during a clinical examination. It has been shown that the use of eye tracking is feasible and deemed to be tolerated by patients (10–11). In a recent study (11) it was also shown that patients with DOC who demonstrated overt and covert tracking eye movements (tracking eye movements detectable with eye tracking only) had better outcome than those without. Although a few earlier studies have also demonstrated promising results (4, 12, 13), there is a paucity of studies incorporating eye-tracking technology into the current gold standard for PDOC assessment: the CRS-R.

The purpose of this study was to investigate if eye tracking can give objective support to the assessment as to whether eye movements not meeting CRS-R criteria are in fact related to instructions and stimuli presented to the patient, and thereby can represent a manifestation of a conscious response. Additionally, the eye-tracking findings were compared with a simultaneously performed clinical assessment. The hypothesis was that voluntary eye movements would be detected more frequently with the eye tracker than during a standard clinical assessment using the CRS-R criteria.

## METHODS

Twelve outpatients with prolonged disorders of consciousness (PDOC) were recruited consecutively as a convenience sample from the rehabilitation clinic of a regional rehabilitation unit in Stockholm, Sweden, from November 2018 to November 2022. The cohort includes 2 patients from a previous publication (10). The examinations and tests were performed in the outpatient clinic or hospital ward. A physician (AKG, specialist in rehabilitation medicine) with experience with patients with PDOC, performed initial screening with regard to inclusion and exclusion criteria. Inclusion criteria were: (*i*) adult patients aged 18–65 years who had had an acquired brain injury in adulthood; and (*ii*) a suspected PDOC. A PDOC was suspected when the patient was unable to demonstrate functional object use and there was an absence of functional communication more than 4 weeks postinjury, but not if aphasia was clinically considered to be the primary reason for this (e.g., dominant hemisphere stroke). PDOC was confirmed by CRS-R assessment. Exclusion criteria were known blindness or deafness, eye disease or severe eye motility restrictions, medical instability, sedation, medical restrictions on neck movements, and if relatives opposed the patient’s participation in the study.

### Consent

The study was approved by the Swedish Ethical Review Authority (Dnr: 2018/150-31). Patients with PDOC cannot give informed consent as, by definition, they lack the capacity to do so. Thus, the patient’s relatives were informed about the study and gave assent for inclusion.

### Equipment

Eye movements were recorded with a wearable eye tracker (Tobii Pro Glasses 2, www.tobiipro.com) where the sensors and scene camera are built into a spectacle frame. The patient wore the eye tracker during the clinical assessment and thereby allowed simultaneous recording of eye movements. The eye tracking system normally applies a 2-eyed calibration procedure to map gaze position (where both eyes look) relative to the frame. The patient is asked to look at a target while the eye tracking system acquires positional data on where the eyes look relative to the scene camera. An inherent challenge when working with patients with PDOC is that compliance with instructions cannot be expected. This becomes obvious when it comes to calibration as it requires an intentional response from the patient, i.e., to fixate the gaze on a small target for several seconds while the eye tracking system acquires data. Furthermore, alternating strabismus is common among these patients, and this complicates or hinders the calibration procedure because it relies on both eyes simultaneously fixating the target. Calibration was subsequently not possible for some patients due to strabismus or inability to fixate on the calibration target. In those cases, the relative change in gaze direction for one eye was used, that is, the change in gaze direction relative to the baseline direction.

### Coma Recovery Scale Revised and procedure

The test paradigm included 4 items from the Coma Recovery Scale Revised (CRS-R) and was performed in a fixed order in the following sequence: object recognition, visual pursuit, fixation, and localization to sound. Each item was subdivided into 4 or 8 trials ending with a total of 24 trials per patient (total 288 trials for all patients) (Table I). The clinical assessment was performed according to the manual and the stated fixed time intervals required for each trial. Careful attempts were made to optimize wakefulness prior to and during testing by tactile and sound stimulation.

**Table I:** Coma Recovery Scale Revised (CRS-R) test items

CRS-R sub-scale	Test item	Number of trials	Method as described in the CRS-R manual
Visual function scale	Object recognition	4 trials as follows: ball left, ball right, cup left, cup right	Present 2 common objects simultaneously and approximately 16 inches apart within the patient’s field of view. Ask the patient to look at the object named (i.e., “Look at the [name object]”). Next, reverse the positions of the 2 objects and ask the patient to look at the same object again (i.e., “Look at the [name object]”). Administer 2 additional trials using the same 2 objects and repeat the above procedure with instruction to look at the other object on both trials. Two trials per object should be administered for a total of 4 trials
	Visual pursuit	8 trials as follows: left, right, left, right, up, down, up, down	Hold a mirror 4–6 inches directly in front of the patient’s face and verbally encourage the patient to fixate on the mirror. Tilt the mirror slowly 45° to the right and left of the vertical midline and 45° above and below the horizontal midline. Repeat the above procedure so that a total of 2 trials are administered in each hemispace (i.e., twice up, twice down, twice left and twice right)
	Fixation	8 trials as follows: left, right, left, right, up, down, up, down	Present a brightly coloured or illuminated object 6–8 inches in front of the patient’s face and then rapidly move to upper, lower, right and left visual fields, respectively, for a total of 4 trials
Auditory function scale	Localization to sound	4 trials as follows: right, left, right, left	Standing behind the patient and out of view, present an auditory stimulus (e.g., patient’s name, voice, noise) from the right side for 5 s. Perform a second trial presenting the auditory stimulus from the left side. Repeat above procedure for a total of 4 trials, 2 on each side. If needed, reorient the head to midline between trials

### Analysis of eye-tracking data

**Fig. 1:**
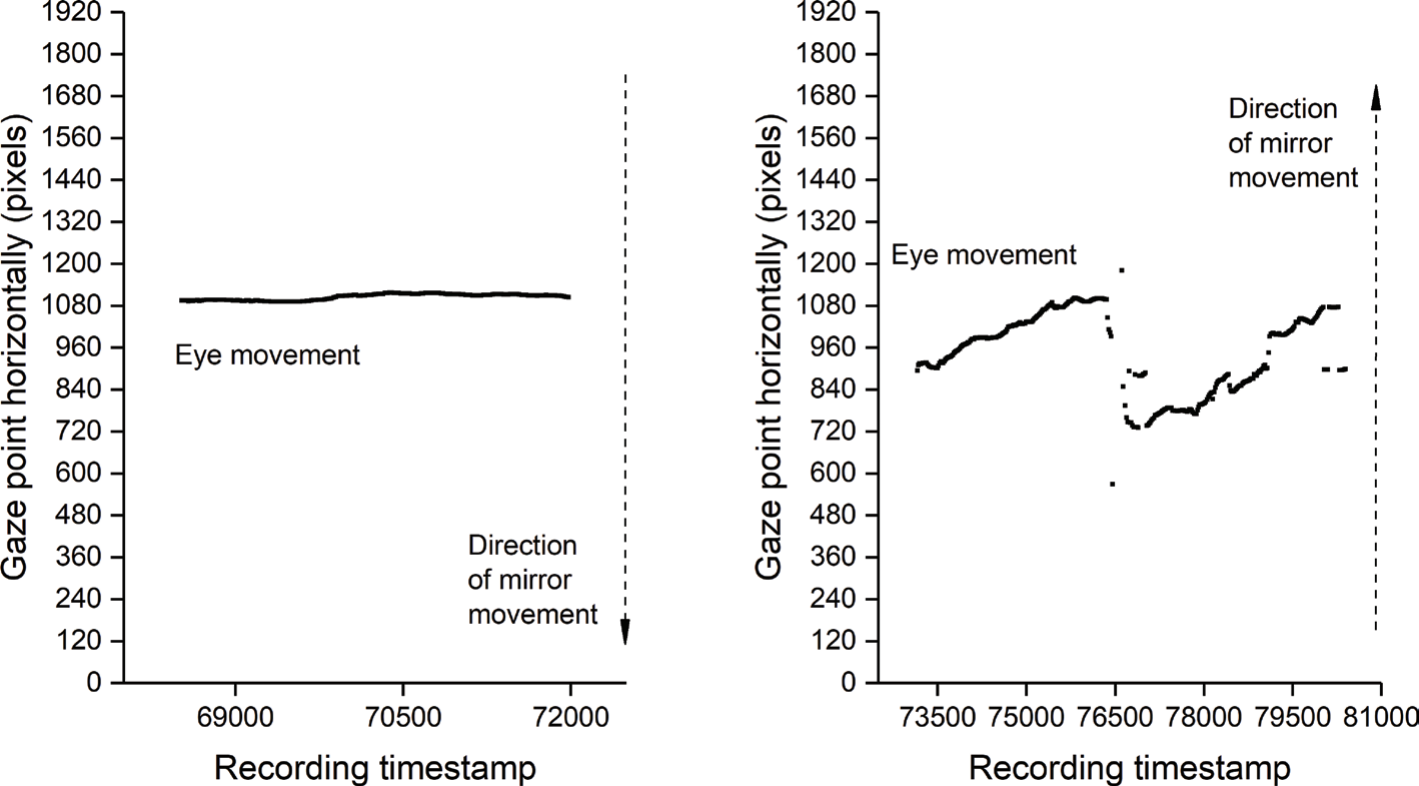
Sample images of plotted eye-movement data for test item “visual pursuit”. The horizontal axis shows the time stamp. The vertical axis represents the eye position relative to the coordinate system of the scene camera measured in pixels. A lower pixel value means the gaze point is towards the left and a higher value means that the gaze point is towards the right. The left image is an example of a trial with no response. The mirror is moved towards the patient’s left-hand side but there is no trend in gaze point data suggesting a response. The right image shows a trial where the mirror is moved to patient’s right-hand side. The gaze point data shows a trend towards higher pixel values, interrupted midway by a re-bound eye movement.

The clinical assessors (IEM and MP) were both experienced licenced clinical neuropsychologists. They had both been trained to perform CRS-R assessments as they work daily with patients with PDOC and perform diagnostic assessments as routine clinical practice. Both assessors were blinded to the eye tracker results during assessment and scoring. Likewise, the scorer of the eye tracking data (JJ) was blinded to the results from the clinical data when scoring and analysing. The data entry was managed by a separate person (KF). Eye-movement data were analysed trial by trial. The Tobii Pro Lab software (https://www.tobii.com/products/software/behavior-research-software/tobii-pro-lab) was used to replay and inspect the recording. The stimulus was time-locked to the eye-tracking system in the following way. The video from the scene camera was replayed and each stimulus was marked in the recording as an event, for example Stimulus Start and Stimulus Stop. The time between Stimulus Start and Stimulus Stop was defined as a Time of Interest and subsequently extracted and exported for analysis. Judgement as to whether the patient made a response or not was based on visual inspection of plotted eye-movement data corresponding to the time window when the patient was exposed to the stimulus. If it was possible to perform calibration, then eye-position data relative to the scene camera coordinate system was used. An example is presented in Fig. 1. If calibration was not possible, then the relative change in gaze direction was used. Further details of the procedure can be found in our previous publication (10).

### Statistical analysis

A Cohen’s kappa analysis was run to determine whether there was agreement between the 2 assessment methods. It should, however, be noted that poor agreement was expected as the aim of this study was to identify a method more likely to detect subtle eye movements than the current gold standard. McNemar’s test with continuity correction was run to determine whether the difference in proportion of responses between assessment methods was statistically significant.

## RESULTS

Twelve patients (median age 43 years (range 18–64 years) were included in the study (Table II). The median time since injury was 3 years 7 months (range 1 month to 9 years 9 months).

**Table II:** Demographic data

Patient	Age, years	Time since injury, years:months	Aetiology	CRS-R total score	Classification according to CRS-R
1	32	9:9	TBI	5	UWS/VS
2	30	4:5	TBI	10	MCS-
3	42	4:5	SAH	10	MCS-
4	55	6:5	SAH	7	UWS/VS
5	50	0:10	Anoxic	7	UWS/VS
6	20	4:0	Anoxic	6	UWS/VS
7	43	3:5	TBI	5	UWS/VS
8	59	3:8	ICH	6	UWS/VS
9	64	2:8	SAH	16	MCS+
10	18	2:2	TBI	12	MCS+
11	53	1:7	SAH	7	UWS/VS
12	35	0:7	TBI	8	MCS-

TBI: traumatic brain injury; SAH: subarachnoid haemorrhage; Anoxic: anoxic brain injury; ICH: intracranial haemorrhage; UWS/VS: unresponsive wakefulness syndrome/vegetative state; MCS: minimally conscious state.

Response data were obtained from 238 out of 288 trials (Table III). No data were obtained for 50 trials. The reason was loss of eye-tracking signal due to closed eyes or eyes pointing in an extreme angle to the side where the eye-tracking system can no longer capture the position and shape of the pupil and the infrared reflection. Eye-tracking data were obtained in median 89.6% of the trials (37.5–100%). Overall, the eye-tracking assessment judged 110 out of 238 trials (46.2%) as a response while the clinical assessment judged 43 out of 238 trials (18.1%) as a response.

**Table III:** Number of trials judged as no response vs response

Test item	Method	No response	Response	Number of trials (total), %
Object recognition	Clinical	30 (71.4%)	12 (28.6%)	42 (48), 87.5%
	Eye tracking	30 (71.4%)	12 (28.6%)	
Visual pursuit	Clinical	66 (84.6%)	12 (15.4%)	78 (96), 81.2%
	Eye tracking	23 (29.5%)	55 (70.5%)	
Fixation	Clinical	68 (86.1%)	11 (13.9%)	79 (96), 82.3%
	Eye tracking	48 (60.7%)	31 (39.3%)	
Localization to sound	Clinical	31 (79.5%)	8 (20.5%)	39 (48), 81.2%
	Eye tracking	27 (69.2%)	12 (30.8%)	
All test items	Clinical	195 (81.9%)	43 (18.1%)	238 (288), 82.6%
	Eye tracking	128 (53.8%)	110 (46.2%)	

A Cohen’s kappa analysis was run to determine whether there was agreement between the 2 assessment methods on whether the patients attempted to comply with the instruction (response) or not (no response). There was agreement on 24 trials being judged as a response and 109 trials being judged as no response. However, the clinical assessment rated 86 trials as no response when the eye-tracking assessment rated them as response, and the clinical assessment rated 19 trials as response when the eye-tracking assessment rated them as no response. There was poor agreement between the method’s judgements (K = 0.073; 95% CI, –0.031 to 0.177; *p* = 0.163). Agreement on a test-by-test basis is described in Table IV. Weak agreement was observed for the test item Object recognition.

**Table IV:** Agreement by test item

Test item			Eye tracking		Total	Statistics
			No response	Response		
Object recognition	Clinical	No response	24	6	30	K = 0.3 (95% CI –0.014–0.614)
		Response	6	6	12	*p* = 0.052
		Total	30	12	42	
Visual pursuit	Clinical	No response	20	46	66	K = 0.022 (95% CI –0.088–0.132)
		Response	3	9	12	*p* = 0.711
		Total	23	55	78	
Visual fixation	Clinical	No response	42	26	68	K = 0.041 (95% CI –0.139–0.221)
		Response	6	5	11	*p* = 0.649
		Total	48	31	79	
Localization to sound	Clinical	No response	23	8	31	K = 0.204 (95% CI –0.119–0.527)
		Response	4	4	8	*p* = 0.186
		Total	27	12	39	

McNemar’s test with continuity correction was also run to determine whether there was a difference between clinical and eye-tracking assessment with regard to the proportion of trials where the patient was deemed to have responded to the prompt. The number of trials where the patient was deemed to have responded was 110 out of 238 with eye-tracking assessment (46.2%) and 43 out of 238 with the clinical assessment (18.1%), a statistically significant difference: χ^2^(1) = 41.486, *p* < 0.001. Analysis by test item is reported in Table V. The eye tracking assessment found a higher proportion of responses for test items “visual pursuit” and “visual fixation”.

**Table V:** Difference between clinical and eye-tracking assessment with regard to the proportion of trials where the patient was deemed to respond to the prompt

Test item	Trials *n*	Trials with deemed response, *n* (%)		Statistics
		Eye tracking	Clinical	
Object recognition	42	12 (28.6%)	12 (28.6%)	Not significant
Visual pursuit	78	55 (70.5%)	12 (15.4%)	χ^2^(1) = 36, *p* < 0.001
Visual fixation	79	31 (39.2%)	11 (13.9%)	*p* < 0.01†
Localization to sound	39	12 (30.8%)	8 (20.5%)	Not significant

† The exact *p*-value was computed based on the binomial distribution because there were 25 or fewer records in the discordant pairs.

## DISCUSSION

The purpose of this study was to investigate if eye tracking can support detection of covert voluntary eye movements and to compare these findings with a simultaneously performed clinical assessment according to the CRS manual criteria.

The outcome of the CRS-R testing differed depending on assessment method. Overall, the eye-tracking assessment judged a significantly higher percentage of trials as a response (46.2%) compared with the clinical assessment (18.1%). These findings were mainly found in test items “visual pursuit” and “visual fixation”. Cohen’s kappa analysis showed poor agreement between the methods, thereby suggesting that assessment with the support of eye tracking gave different results from clinical judgement alone.

One inherent problem is that there is no absolute gold standard for assessment of conscious responses. CRS-R provides a useful standardization for clinical assessment with necessarily fairly coarse criteria for decisions on whether responses are related to scale items or not, emphasizing exclusion of responses that could be non-stimuli related due to long latency or short duration. These difficulties are not completely solved by eye tracking: we cannot be absolutely confident that a positive eye-tracking response is truly positive (i.e., that it represents a sign of consciousness), and that a negative response is truly negative, as no definitive answer exists. Consequently, typical sensitivity and specificity analyses are not very informative. One way to handle this is by investigating whether eye-tracking responses are more predictive of outcome than clinical judgements. In an eye-tracking study on patients with DOC in the subacute phase, patients who demonstrated tracking eye movements (both covert and overt) had a better outcome than those who did not (11). However, this was a small study in need of replication. Our study included patients with prolonged disorders of consciousness. The median time after injury was more than 3 years, from which point improvement is unlikely. It may be that patients in a subacute stage respond differently, as has been shown in another research study (11). Also, if the examination had been carried out at an earlier stage, it would have been possible to evaluate whether the eye-tracking method had a better prognostic value than an ordinary method as a follow-up study would have been of interest. On the other hand, as in several cases there were indications of conscious responses that was not detected earlier, the method can give indication of a higher level of awareness in some of the patients than today’s clinical methods can confirm.

### Study limitations

Some study limitations need to be considered. The sample size was low and included only patients with prolonged disorders of consciousness. This limits to what extent the findings can be generalized to the PDOC patient group.

Methodological limitations need to be considered. The recorded eye movements were in many cases subtle, strained, and frequently interrupted by closed eyes, rebound eye movements, or alternating fixation in the presence of strabismus. This makes clinical assessment through observation very challenging. On the plus side, the eye-tracking technology may serve as a useful way of documenting observations and offer a base for review and discussions in the medical and rehabilitation team. This is especially relevant given that fatiguability of patients often means that simply repeating the testing may not confirm the original finding. On the other hand, suboptimal recorded eye-movement data, problems related to calibration, and subsequent difficulties linking eye movements to the stimuli makes it very challenging to design a reliable automated procedure. Based on the findings in this and previous studies, eye tracking may, however, serve as a useful tool that may complement observation (10).

The wearing of the eye tracker appeared to be generally well accepted by the patients as per previous observations (10). However, 4 patients showed poor responses when wearing the eye tracker but upon re-testing without the eye tracker they showed partially improved responses. Based on this observation it cannot be excluded that the eye tracker in its current form may make some patients uncomfortable, which in turn could affect the outcome of the assessment. Consequently, if using an eye tracker in clinical practice, the assessments should also be done without the eye tracker from time to time.

When scoring the eye-tracking data, the CRS-R criteria were not followed. Thus, some of the responses identified only with the eye tracker could also have been recorded on a clinical exam, if the response criteria had been less strict. The CRS-R criteria have been developed for clinical examinations and it is our understanding that many of these (e.g., the duration of a visual fixation) have been constructed to minimize the risk of random movements/fixations/responses incorrectly being counted as responses. We consider that this risk is reduced when using an eye tracker and that some of the original CRS-R criteria therefore are redundant. With conservative criteria, the risk of false negatives increases, which can be critical for the patient assessed. In our opinion, the great advantage of eye tracking is the reduced risk of these false negatives.

Also, it is not inconceivable that the scoring during the clinical examination may have been affected by the fact that the patient wore glasses, as these could possibly obscure the patient’s eyes and complicate the assessment of the eye movements.

### Future development and research

Many patients sat in an adapted position due to consequences of their brain injury. This made it a bit more difficult to wear the eye-tracking equipment in the intended way and some patients appeared to be uncomfortable when wearing it. This occurred despite advanced seating solutions being incorporated into local rehabilitation processes. To make eye tracking a clinically useful method, the design of the glasses needs to be improved to increase comfort for the patient as well as allowing recording of larger visual angles.

To make the method clinically useful, the analysis methods also need to be improved. The current Tobii analysis program assumes that it is the eyes that move, and the surroundings are still. Analysing the heat map only works if the patient’s eyes can be calibrated, which is not possible in severe strabismus. In cases where heat maps are not possible the analysis is far too time-consuming to be practical.
